# Antimicrobial Photodynamic Inactivation Using Riboflavin 5′-Phosphate and a 450 nm Diode Laser: An In Vitro Dose-Optimisation Study

**DOI:** 10.3390/pharmaceutics18080977

**Published:** 2026-08-08

**Authors:** Maciej Łopaciński, Anna Mertas, Anna Kuśka-Kiełbratowska, Elżbieta Bobela, Eleftherios Terry R. Farmakis, Dariusz Skaba, Rafał Wiench

**Affiliations:** 1 Department of Periodontal Diseases and Oral Mucosa Diseases, Faculty of Medical Sciences in Zabrze, Medical University of Silesia, 40-055 Katowice, Poland; anna.kuska-kielbratowska@sum.edu.pl (A.K.-K.); dskaba@sum.edu.pl (D.S.); 2 Department of Microbiology and Immunology, Faculty of Medical Sciences in Zabrze, Medical University of Silesia, 40-055 Katowice, Poland; amertas@sum.edu.pl (A.M.); ebobela@sum.edu.pl (E.B.); 3 Department of Endodontics, School of Dentistry, National and Kapodistrian University of Athens, 2 Thivon Street, Goudi, 11527 Athens, Greece; elefarm@dent.uoa.gr

**Keywords:** antimicrobial photodynamic therapy, riboflavin 5′-phosphate, 450 nm diode laser, *Candida*, *Staphylococcus aureus*, *Enterococcus faecalis*, dose optimization, reactive oxygen species

## Abstract

**Background**: Rising antifungal and antibiotic resistance among *Candida* species, *Staphylococcus aureus*, and *Enterococcus faecalis* has renewed interest in antimicrobial photodynamic therapy (aPDT) as a resistance-independent strategy. Riboflavin 5′-phosphate is a biocompatible, blue-light-activated photosensitizer, but standardized dosing across fungal and bacterial targets is lacking. **Objective**: The aim of this study was to systematically optimize pre-irradiation incubation time, photosensitizer volume, irradiation time, and laser power for riboflavin 5′-phosphate aPDT (450 nm diode laser) against *C. albicans*, *C. glabrata*, *C. krusei*, *S. aureus*, and *E. faecalis*, and compare species susceptibility under optimized conditions. Methods: ATCC strains were treated with 0.1% riboflavin 5′-phosphate across four groups (photodynamic, photosensitizer-only, laser-only, control) in a staged design optimizing incubation (1–30 min), photosensitizer volume (50–150 µL), irradiation time (10–120 s), and power (50–400 mW). Viable counts (CFU/mL) were quantified. **Results**: Significant reductions occurred only with combined light-plus-photosensitizer treatment. Optimal parameters were 15 min incubation, 100 µL photosensitizer for *Candida* spp. (50 µL for bacteria), and 120 s at 400 mW, though *C. albicans* and *C. krusei* plateaued by 60 s. Maximum reductions were modest: 53.5% (*C. albicans*), 46.7% (*S. aureus*), 37.9% (*C. glabrata*), 35.9% (*C. krusei*), and 26.5% (*E. faecalis*), all below 1 log_10_. A significant light × photosensitizer interaction, confirming photodynamic specificity, was seen for *C. albicans*, *C. glabrata*, and *S. aureus*, but not *C. krusei* or *E. faecalis*. **Conclusions**: Riboflavin 5′-phosphate aPDT under 450 nm light produces reproducible, dose-dependent, species-specific antimicrobial activity, best suited as an adjunctive rather than stand-alone therapy pending biofilm and in vivo validation.

## 1. Introduction

Infections caused by opportunistic microorganisms represent an increasing global health burden, particularly among immunocompromised and critically ill patients. *Candida* species are among the most prevalent fungal pathogens, responsible for a wide spectrum of invasive and mucosal infections [1,2]. Although *Candida albicans* remains the dominant etiologic agent, non-albicans species such as *Candida glabrata* and *Candida krusei* have gained clinical relevance due to intrinsic or acquired resistance to antifungal agents, including azoles [2,3]. Concurrently, bacterial pathogens such as *Staphylococcus aureus* and *Enterococcus faecalis* pose significant therapeutic challenges, particularly in the context of device-associated infections, wound infections, and endodontic disease, where biofilm formation and antimicrobial resistance substantially limit treatment success [1,4]. The rising incidence of both antifungal and antibacterial resistance, combined with limited therapeutic options and the systemic toxicity of available agents, necessitates the development of alternative treatment strategies [1,2,4].

Antimicrobial photodynamic therapy (aPDT) has emerged as a promising non-invasive modality for the management of localized infections caused by a broad range of microorganisms. Its mechanism requires a photosensitizer, light of an appropriate wavelength, and molecular oxygen. Upon activation, the photosensitizer transitions to an excited triplet state and generates reactive oxygen species (ROS) via Type I (electron transfer) and Type II (energy transfer) pathways, producing superoxide radicals, hydrogen peroxide, hydroxyl radicals, and singlet oxygen [5,6,7]. These ROS induce oxidative damage to lipids, proteins, and nucleic acids, resulting in microbial cell death [1,8,9]. Unlike conventional antimicrobials targeting specific cellular pathways, aPDT exerts multi-target oxidative stress, which significantly reduces the likelihood of resistance development and renders it effective against both fungi and bacteria, including drug-resistant strains [1,8,10].

The efficacy of aPDT is critically dependent on the choice of photosensitizer. Riboflavin (vitamin B_2_) and its derivative riboflavin 5′-phosphate (flavin mononucleotide, FMN) are biocompatible compounds with favorable safety profiles. Riboflavin exhibits strong absorption in the blue spectrum (445–450 nm) and a high singlet oxygen quantum yield (~0.54), while its phosphorylated form offers improved water solubility [11,12,13,14,15]. Riboflavin-mediated aPDT has demonstrated substantial antibacterial efficacy against a range of pathogens, including methicillin-resistant *Staphylococcus aureus* and *Enterococcus faecalis*, achieving marked reductions in viable counts under optimized conditions [14,15,16]. The antimicrobial effect is mediated by both Type I and Type II ROS, with evidence suggesting sustained activity attributable to photolytic by-products [14,15,17]. Antifungal applications of riboflavin-mediated aPDT have also been reported, with available studies demonstrating reductions in *Candida* viability in both planktonic and biofilm forms; however, complete eradication is inconsistent and protocols remain experimentally varied [11]. Comparative susceptibility among *Candida* species is poorly defined despite known differences in antifungal resistance profiles and pathogenicity, and systematic dose-optimization data encompassing both bacterial and fungal targets are lacking [10,18,19].

Light wavelength is a critical determinant of aPDT efficacy. Blue light (400–470 nm) efficiently activates riboflavin due to strong spectral absorption and higher photon energy compared to red light, allowing effective ROS generation at lower doses [20,21]. However, limited tissue penetration (approximately 1–2 mm) restricts its application to superficial and localized infections, including oral candidiasis, denture stomatitis, infected wounds, and endodontic infections [20,22]. A wavelength of 450 nm represents a practical balance between absorption efficiency and penetration depth for such applications [14,15].

The present study aimed to systematically optimize riboflavin 5′-phosphate-mediated aPDT using a 450 nm diode laser against a panel of fungal and bacterial pathogens: *Candida albicans*, *Candida glabrata*, *Candida krusei*, *Staphylococcus aureus*, and *Enterococcus faecalis*. The fungal species were selected based on their clinical prevalence and distinct antifungal susceptibility profiles, while the bacterial species represent common Gram-positive pathogens associated with both community-acquired and healthcare-associated infections. A structured dose-optimization approach was applied, varying pre-irradiation incubation time, photosensitizer volume, irradiation time, and output power. The inclusion of appropriate control groups enabled precise attribution of observed effects to the photodynamic mechanism rather than to either agent alone. This study provides parameter-specific data and comparative susceptibility insights across both fungal and bacterial targets, supporting the development of aPDT as a broad-spectrum adjunctive strategy in the management of superficial and localized microbial infections.

The aims of this study were:

1. To determine the optimal pre-irradiation incubation time of tested microorganisms with riboflavin 5′-phosphate (0.1%) as a photosensitizer prior to laser irradiation.
2. To identify the most effective photosensitizer volume (50, 100, or 150 µL) for maximizing antimicrobial activity.
3. To evaluate the effect of varying laser irradiation time (10, 30, 60, and 120 s) on the efficacy of aPDT against the tested fungal and bacterial species.
4. To evaluate the effect of varying laser output power (50, 100, 200, and 400 mW) on aPDT efficacy.
5. To compare the susceptibility of *C. albicans* ATCC 10231, *C. glabrata* ATCC 66032, *C. krusei* ATCC 14243, *S. aureus* ATCC 29213, and *E. faecalis* ATCC 29212 to aPDT under optimized conditions.
6. To confirm that the antimicrobial effect observed is attributable specifically to the photodynamic reaction, rather than to the photosensitizer or laser light acting independently.

## 2. Materials and Methods

### 2.1. Null Hypothesis

Antimicrobial photodynamic therapy using riboflavin 5′-phosphate as a photosensitizer activated by a 450 nm diode laser does not reduce the number of viable cells of *Candida albicans* ATCC 10231, *Candida krusei* ATCC 14243, *Candida glabrata* ATCC 66032, *Staphylococcus aureus* ATCC 29213, or *Enterococcus faecalis* ATCC 29212 to a greater extent than laser irradiation alone or photosensitizer exposure alone, under any of the tested irradiation or incubation conditions. The *in vitro* experimental studies were conducted at the Microbiological Laboratory of Silesia LabMed, Department of Microbiology and Immunology, Faculty of Medical Sciences in Zabrze, Medical University of Silesia in Katowice.

### 2.2. Standard Microbial Strains

The study was performed using the following standard microbial strains obtained from the American Type Culture Collection (ATCC, Manassas, VA, USA):

• Three standard fungal strains of the genus *Candida*: *Candida albicans* ATCC 10231, *Candida krusei* ATCC 14243, and *Candida glabrata* ATCC 66032;
• Two standard bacterial strains: *Staphylococcus aureus* ATCC 29213 and *Enterococcus faecalis* ATCC 29212.

Standard fungal strains were cultured on Sabouraud agar (bioMérieux SA, Marcy l’Étoile, France), while bacterial strains were cultured on Columbia agar (bioMérieux SA, Marcy l’Étoile, France) supplemented with 5% sheep blood. All cultures were incubated at 37 °C and subcultured every 48–72 h in accordance with ATCC requirements. Microorganisms from 24-h cultures were used for all experiments. Working suspensions were prepared in sterile 0.9% NaCl solution, with density measured using a Densi-La-Meter II laboratory densitometer (Erba Polska Sp. z o.o., Kraków, Poland). Suspension density and volume were adjusted so that the total inoculum delivered to each well was held constant at 6 × 10^7^ CFU/well across all experimental variants (see Section 2.9 and Table 1 for the calculation underlying this target and its adjustment across photosensitizer volumes).

### 2.3. Photosensitizer

The photosensitizer used in all experiments was riboflavin 5′-phosphate sodium salt hydrate (Sigma-Aldrich Co., St. Louis, MO, USA), supplied as a powder. A 0.1% (*w*/*v*) stock solution was prepared in sterile demineralized water immediately prior to use. For comparison, European Pharmacopoeia-grade riboflavin was also tested at its maximum achievable aqueous concentration (0.01%) but produced no measurable antimicrobial effect under any tested condition and was therefore excluded from further experimentation. All photosensitizer solutions were prepared and handled under amber light to prevent premature photoactivation.

### 2.4. Light Source

The light source was a 450 nm diode laser (PIOON S1 Blue, PIOON, Wuhan, China), operating in continuous wave (CW) mode. The laser was fitted with a flat-top applicator tip of approximately 0.5 cm^2^ surface area (8 mm diameter), providing uniform energy distribution across the irradiated area. Laser output power was calibrated prior to each experimental session using a laser power meter. Output power settings of 50, 100, 200, and 400 mW were applied across the experimental stages, combined with irradiation times of 10, 30, 60, and 120 s, yielding energy densities (fluences) ranging from 1.0 to 96.0 J/cm^2^.

### 2.5. Experimental Design and Group Allocation

All experiments employed four parallel experimental groups:

1. (L+P+)—photodynamic treatment group: microbial suspension exposed to both riboflavin 5′-phosphate and laser irradiation;
2. (L−P+)—photosensitizer-only group: suspension exposed to riboflavin 5′-phosphate without laser irradiation;
3. (L+P−)—laser-only group: suspension subjected to laser irradiation without photosensitizer;
4. (L−P−)—untreated control group: suspension without photosensitizer or laser irradiation.

This four-group design was maintained throughout all experimental stages to allow attribution of observed effects to the photodynamic mechanism specifically, controlling for dark toxicity of the photosensitizer and phototoxicity of the laser independently.

### 2.6. General Experimental Procedure

All procedures were performed in darkness at room temperature inside a class II biological safety cabinet (BIO ACTIVA VE 120, AQUARIA SRL, Lacchiarella, Italy). A volume of 200 µL of the working microbial suspension was added to selected wells of 96-well black sterile microtiter plates with lids (Thermo Fisher Scientific, Waltham, MA, USA), leaving one empty well between consecutive samples to prevent light cross-diffusion. Riboflavin 5′-phosphate solution (volume as specified per stage; 50 µL unless otherwise stated) was added to the wells of the L+P+ and L−P+ groups, while an equivalent volume of sterile tryptone water was added to the L+P− and L−P− groups to maintain equal total well volumes. Plates were shaken for 1 min at 350 rpm at 35 °C in a PST-60 HL-4 thermoshaker (Biosan, Riga, Latvia) to ensure uniform photosensitizer distribution, then incubated in darkness for the designated pre-irradiation period.

Following incubation, wells in the L+P+ and L+P− groups were irradiated sequentially, with the laser tip mounted on a stand positioned 1 mm above the plate surface. Non-irradiated wells were covered with a black matte screen containing an aperture matching the applicator tip diameter, to prevent light diffusion to adjacent wells.

Sample processing and CFU/mL calculation.

Immediately after irradiation, a 10 µL aliquot from each well was transferred into a tube containing 4 mL of sterile tryptone water (dilution factor = (4000 µL + 10 µL)/10 µL ≈ 401). From this dilution, 10 µL was plated in duplicate onto the appropriate culture medium. Fungal suspensions (*C. albicans*, *C. krusei*, *C. glabrata*) were plated onto Sabouraud agar and incubated for 48 h at 35 °C; bacterial suspensions (*S. aureus*, *E. faecalis*) were plated onto Columbia agar with 5% sheep blood and incubated for 24 h at 37 °C. Colonies were counted using an automated ProtoCOL 3 colony counter (Synbiosis, Cambridge, UK). Plates yielding countable colonies (approximately 30–300 CFU per plate) were used for calculation. Viable density in the original well suspension was calculated as:

CFU/mL = (N × D)/V

where N = number of colonies counted, D = dilution factor (≈401), and V = volume plated, in mL (0.01 mL). This is equivalent to multiplying the raw colony count by a conversion factor of approximately 4.0 × 10^4^. All viable counts reported in the Results (both tabulated values and figures) represent CFU/mL calculated using this formula, applied uniformly across fungal and bacterial species, to allow direct comparison between raw plate counts and the CFU/mL values shown graphically.

### 2.7. Stage I—Optimization of Pre-Irradiation Incubation Time

Stage I was performed using *Candida albicans* ATCC 10231 as the index organism, with fixed laser parameters (output power: 400 mW; irradiation time: 60 s, corresponding to a fluence of 48 J/cm^2^) and a fixed photosensitizer volume of 50 µL. Six pre-irradiation incubation periods were evaluated: 1, 5, 10, 15, 20, and 30 min, with n = 4 replicates per group at each time point. The incubation period yielding the greatest reduction in viable CFU/mL in the L+P+ group relative to L−P− was selected as the optimal pre-irradiation incubation time and applied uniformly in Stages II and III.

### 2.8. Stage II—Optimization of Photosensitizer Volume

Stage II was performed using the optimal pre-irradiation incubation time established in Stage I, with fixed laser parameters (400 mW, 60 s) held constant to isolate the effect of photosensitizer volume. Three volumes of riboflavin 5′-phosphate solution were evaluated, 50, 100, and 150 µL, with n = 6 replicates per group. To maintain a constant inoculum of 6 × 10^7^ CFU/well across all volume variants, the volume and density of the working microbial suspension were adjusted accordingly (Table 1).

Because fungal and bacterial cells may differ in photosensitizer uptake kinetics and cell envelope permeability, this optimization was performed independently in two index organisms: *Candida albicans* ATCC 10231 (representing the fungal panel) and *Staphylococcus aureus* ATCC 29213 (representing the bacterial panel). The volume yielding the greatest reduction in each index organism was subsequently applied to all species within that kingdom (i.e., all three *Candida* species and both bacterial species, respectively) in Stage III.

### 2.9. Stage III—Optimization of Laser Irradiation Parameters

Stage III was performed using the optimal pre-irradiation incubation time (Stage I) and the optimal photosensitizer volume established for each kingdom in Stage II (*Candida* species: volume established in the *C. albicans* index experiment; bacterial species: volume established in the *S. aureus* index experiment). All five microbial species were evaluated in this stage, with all four experimental groups (L+P+, L−P+, L+P−, L−P−) included throughout.

Two irradiation parameters were evaluated independently, with n = 6 replicates per group:

• Irradiation time: at a fixed output power of 400 mW, four irradiation durations were tested—10, 30, 60, and 120 s, yielding fluences of 8, 24, 48, and 96 J/cm^2^, respectively.
• Laser output power: at a fixed irradiation time of 120 s, four output power levels were tested—50, 100, 200, and 400 mW, yielding fluences of 12, 24, 48, and 96 J/cm^2^, respectively.

### 2.10. Statistical Analysis

Data are expressed as mean ± standard deviation (SD) of CFU/mL, calculated as described in Section 2.6. Between-group comparisons were performed using independent-samples Student’s *t*-tests. The Levene F-test for equality of variances was applied prior to each comparison, with Welch’s correction used where variances were unequal. A significance threshold of *p* < 0.05 was applied throughout. Pairwise comparisons of L+P+ group means across irradiation time points and power levels were performed to assess dose–response relationships. All statistical analyses were performed using Statistica version 13.3 (TIBCO Software Inc., Palo Alto, CA, USA).

## 3. Results

### 3.1. Stage I—Optimization of Pre-Irradiation Incubation Time

The effect of pre-irradiation incubation time with 0.1% riboflavin 5′-phosphate was evaluated for *Candida albicans* ATCC 10231 using fixed irradiation parameters (450 nm diode laser, 400 mW, 60 s, corresponding to a fluence of 48 J/cm^2^; 50 µL photosensitizer). Six incubation periods (1, 5, 10, 15, 20, and 30 min) were investigated. Untreated control (L−P−) CFU/mL values varied modestly across the six independent experimental runs (range: 2.72 × 10^6^–3.48 × 10^6^ CFU/mL), reflecting normal batch-to-batch variability in inoculum preparation across separately processed plates rather than any biological effect of incubation time on the untreated control itself. Across all incubation periods, the L+P+ group showed reduced CFU/mL relative to its matched control. The numerically greatest reduction was observed at 15 min (38.2%; 0.209 log_10_), followed by 30 min (36.3%; 0.196 log_10_) and 10 min (32.8%; 0.173 log_10_); reductions at 1, 5, and 20 min were smaller (24.2%, 18.9%, and 14.4%, respectively; 0.120, 0.091, and 0.067 log_10_). A one-way ANOVA comparing L+P+ CFU/mL across the six incubation times did not reach overall statistical significance (F(5,12) = 1.82, *p* = 0.183), and pairwise comparisons of each L+P+ group against its matched control reached significance only at 10 min (*p* = 0.021), 15 min (*p* = 0.0047), and 30 min (*p* = 0.0024). The reduction achieved at 10 min was numerically close to that achieved at 15 min (32.8% vs. 38.2%, a difference of under 0.04 log_10_), indicating that the two incubation times produce comparable antifungal activity within this dataset. The 15 min incubation interval, which produced the numerically greatest reduction, was applied to all five microbial species in Stages II and III below (Table 2).

### 3.2. Stage II—Optimization of Photosensitizer Volume

Photosensitizer volume was optimized using the 15 min incubation time established in Stage I, at fixed irradiation parameters (400 mW, 60 s). This stage was performed in *C. albicans* ATCC 10231 as the fungal index organism, testing 50, 100, and 150 µL of riboflavin 5′-phosphate solution (n = 6 replicates per group). CFU/mL in the L+P+ group was lowest at 100 µL photosensitizer volume (1.86 × 10^6^ ± 2.80 × 10^5^ CFU/mL; control 4.00 × 10^6^ ± 3.70 × 10^5^ CFU/mL; 53.5% reduction, 0.333 log_10_), compared with 44.5% (0.256 log_10_) at 50 µL (treated: 1.79 × 10^6^ ± 2.90 × 10^5^; control 3.23 × 10^6^ ± 4.14 × 10^5^) and 46.8% (0.274 log_10_) at 150 µL (treated: 2.27 × 10^6^ ± 4.80 × 10^5^; control 4.26 × 10^6^ ± 6.80 × 10^5^). A one-way ANOVA across the three volumes did not reach statistical significance (F(2,15) = 2.05, *p* = 0.163), indicating that although 100 µL produced the numerically greatest reduction, the three tested volumes did not differ robustly from one another under this sample size. While a full three-volume comparison was not systematically replicated for the bacterial species, preliminary range-finding tests in *S. aureus* indicated that increasing the photosensitizer volume did not yield further reductions in viable counts, consistent with the volume-independent pattern systematically observed in *C. albicans*. On this basis, 50 µL was adopted as the standard working volume for both bacterial species in Stage III. We acknowledge, however, that the volume optimisation for the bacterial strains was based on limited preliminary observations rather than the full systematic comparison performed for *C. albicans*, and this constitutes a methodological limitation of the present study. The volume identified as optimal for *C. albicans* (100 µL) was applied to all three *Candida* species in Stage III; 50 µL was applied to both bacterial species (Table 3).

### 3.3. Stage III—Optimization of Laser Irradiation Parameters

Stage III used the 15 min incubation time (Stage I) and the kingdom-specific photosensitizer volumes established in Stage II (100 µL for *Candida* spp.; 50 µL for bacterial spp.), evaluating all five species across four irradiation times (10, 30, 60, 120 s at fixed 400 mW) and four power levels (50, 100, 200, 400 mW at fixed 120 s). All four experimental groups were included at every condition. Across conditions, two-way ANOVA (light × photosensitizer) confirmed that neither the photosensitizer alone (L−P+) nor the laser alone (L+P−) produced meaningful reductions relative to L−P− controls in any species, while a significant light × photosensitizer interaction emerged for the majority of conditions (Table 4), supporting a photodynamic-specific mechanism rather than dark toxicity or photothermal injury.

#### 3.3.1. Effect of Irradiation Time

**Figure 1:**
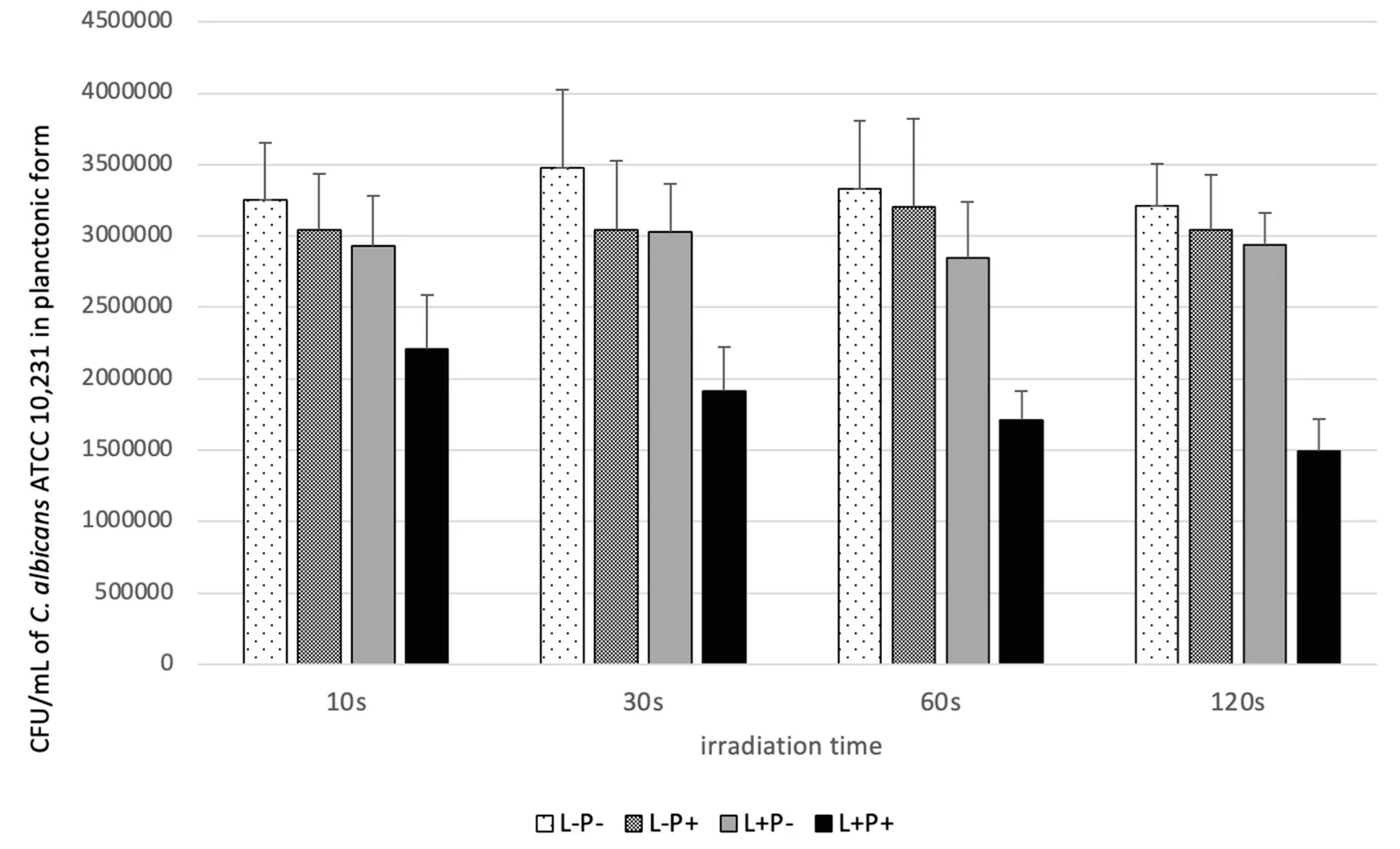
CFU/mL of planktonic *C. albicans* ATCC 10,231 after laser only (L+P−), photosensitizer only (L−P+), both (L+P+), or neither (L−P−), across irradiation times of 10–120 s, showing the greatest reduction with combined treatment.

*Candida albicans*. At 400 mW, CFU/mL in L+P+ declined progressively with irradiation time (32.2%, 45.0%, and 48.6% reduction at 10, 30, and 60 s; 0.169, 0.260, and 0.289 log_10_), reaching a maximum of 53.5% (0.333 log_10_) at 120 s. The light × photosensitizer interaction became significant from 60 s onward (F(1,20) = 7.61, *p* = 0.012 at 60 s; F(1,20) = 29.51, *p* < 0.0001 at 120 s), while the interaction did not reach significance at 10 or 30 s (Figure 1).

**Figure 2:**
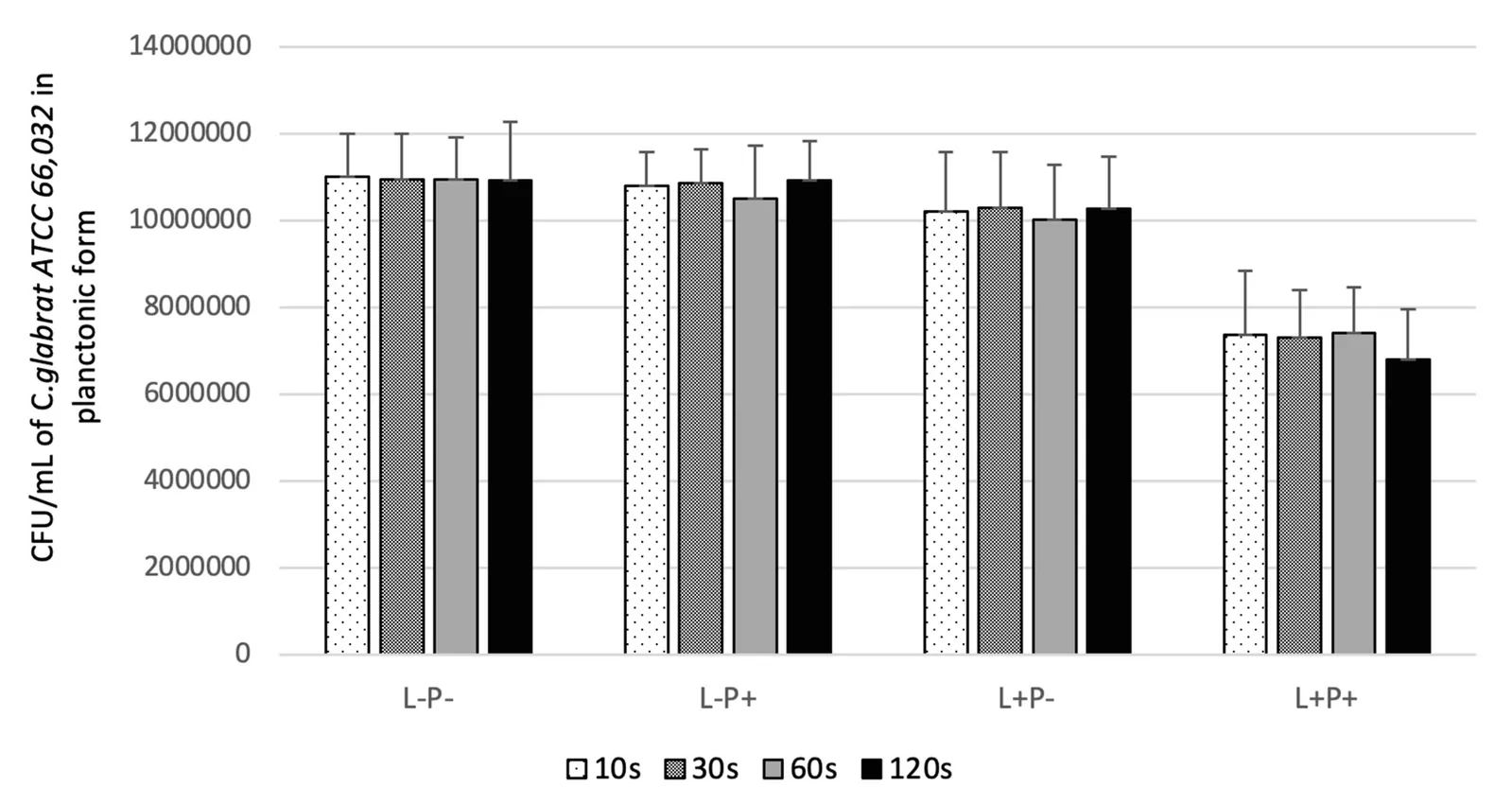
CFU/mL of planktonic *C. glabrata* ATCC 66,032 across irradiation times (10–120 s) for L−P−, L−P+, L+P−, and L+P+ groups, showing a marked reduction only in the combined L+P+ group, largely independent of irradiation time.

*Candida glabrata*. Control CFU/mL was substantially higher than for *C. albicans* (range 1.09 × 10^7^–1.10 × 10^7^ CFU/mL). Reductions were similar across 10, 30, and 60 s (33.2%, 33.2%, and 32.3%; 0.175, 0.175, and 0.169 log_10_) and reached a maximum of 37.9% (0.207 log_10_) at 120 s. The light × photosensitizer interaction was significant at every time point tested (Figure 2).

**Figure 3:**
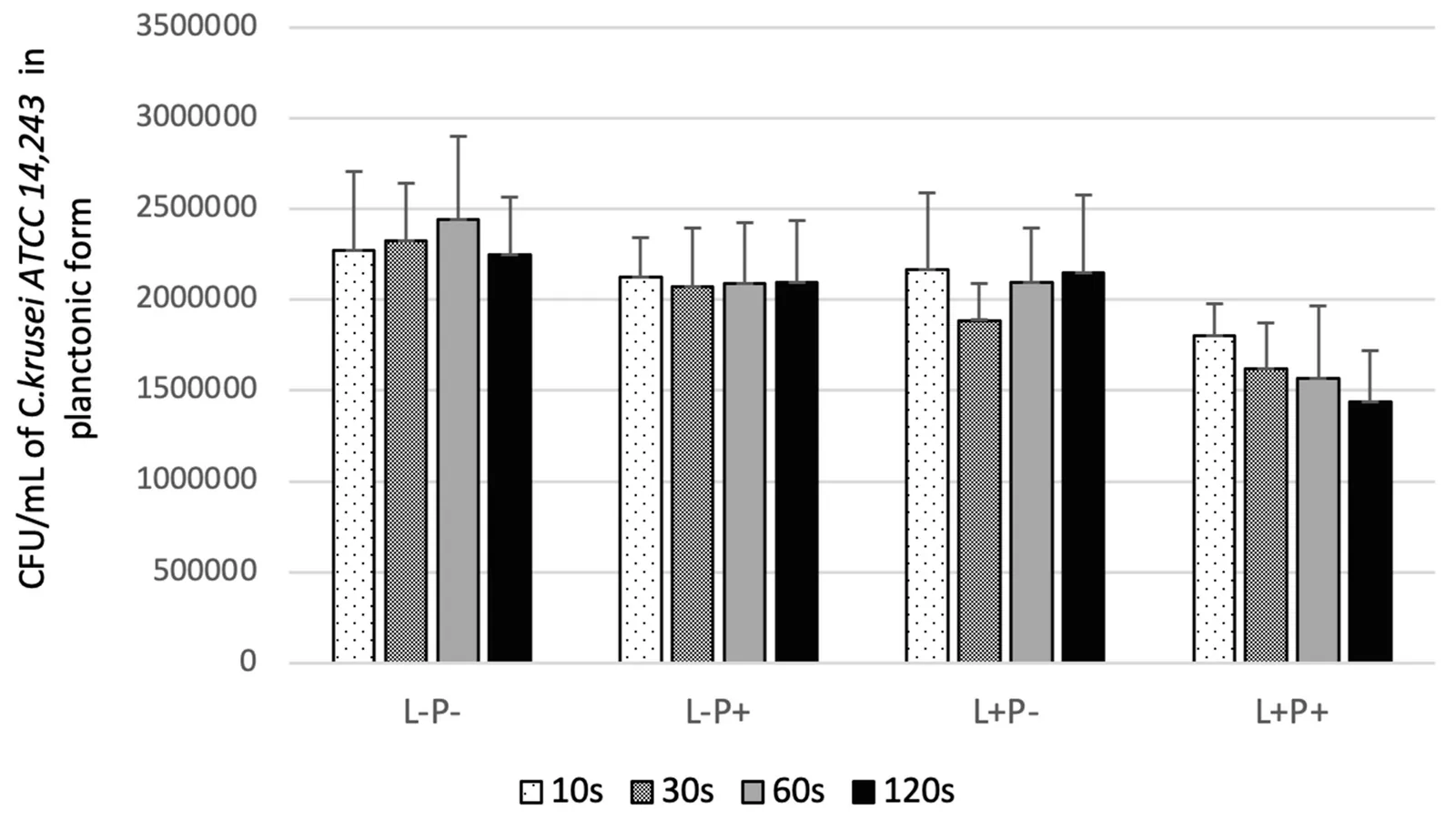
CFU/mL of planktonic *C. krusei* ATCC 14,243 across irradiation times (10–120 s) for L−P−, L−P+, L+P−, and L+P+ groups, showing the lowest counts in the combined L+P+ group, with a modest further decline at longer irradiation times.

*Candida krusei*. Baseline control CFU/mL ranged from 2.25 × 10^6^ to 2.44 × 10^6^ across conditions. Reductions increased progressively with irradiation time, from 20.8% (0.101 log_10_) at 10 s to 35.9% (0.193 log_10_) at 120 s, though the light × photosensitizer interaction term did not reach statistical significance at any individual time point (all *p* > 0.06; Table 4), indicating that, unlike in *C. albicans* and *C. glabrata*, the combined treatment effect in *C. krusei* was not clearly distinguishable from the additive contributions of light and photosensitizer alone under the two-way model (Figure 3).

**Figure 4:**
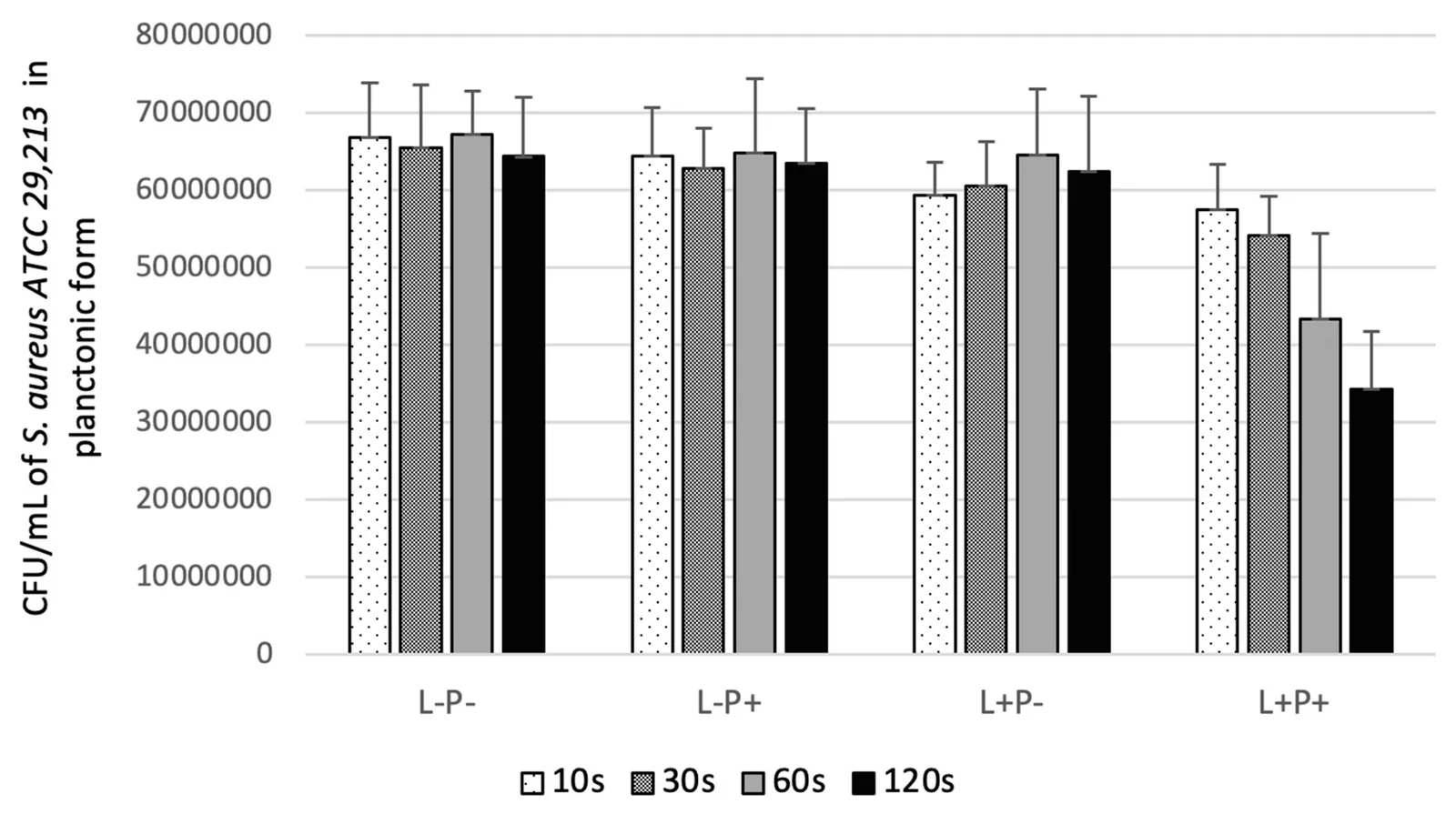
CFU/mL of planktonic *S. aureus* ATCC 29,213 across irradiation times (10–120 s) for L−P−, L−P+, L+P−, and L+P+ groups, showing a progressive reduction in the L+P+ group with increasing irradiation time, most pronounced at 120 s.

*Staphylococcus aureus***.** Reductions increased progressively with irradiation time, from 13.8% (0.065 log_10_) at 10 s to 46.7% (0.274 log_10_) at 120 s. The light × photosensitizer interaction reached significance at 60 s (F(1,20) = 6.67, *p* = 0.018) and 120 s (F(1,20) = 17.14, *p* = 0.0005), but not at 10 or 30 s (Figure 4).

**Figure 5:**
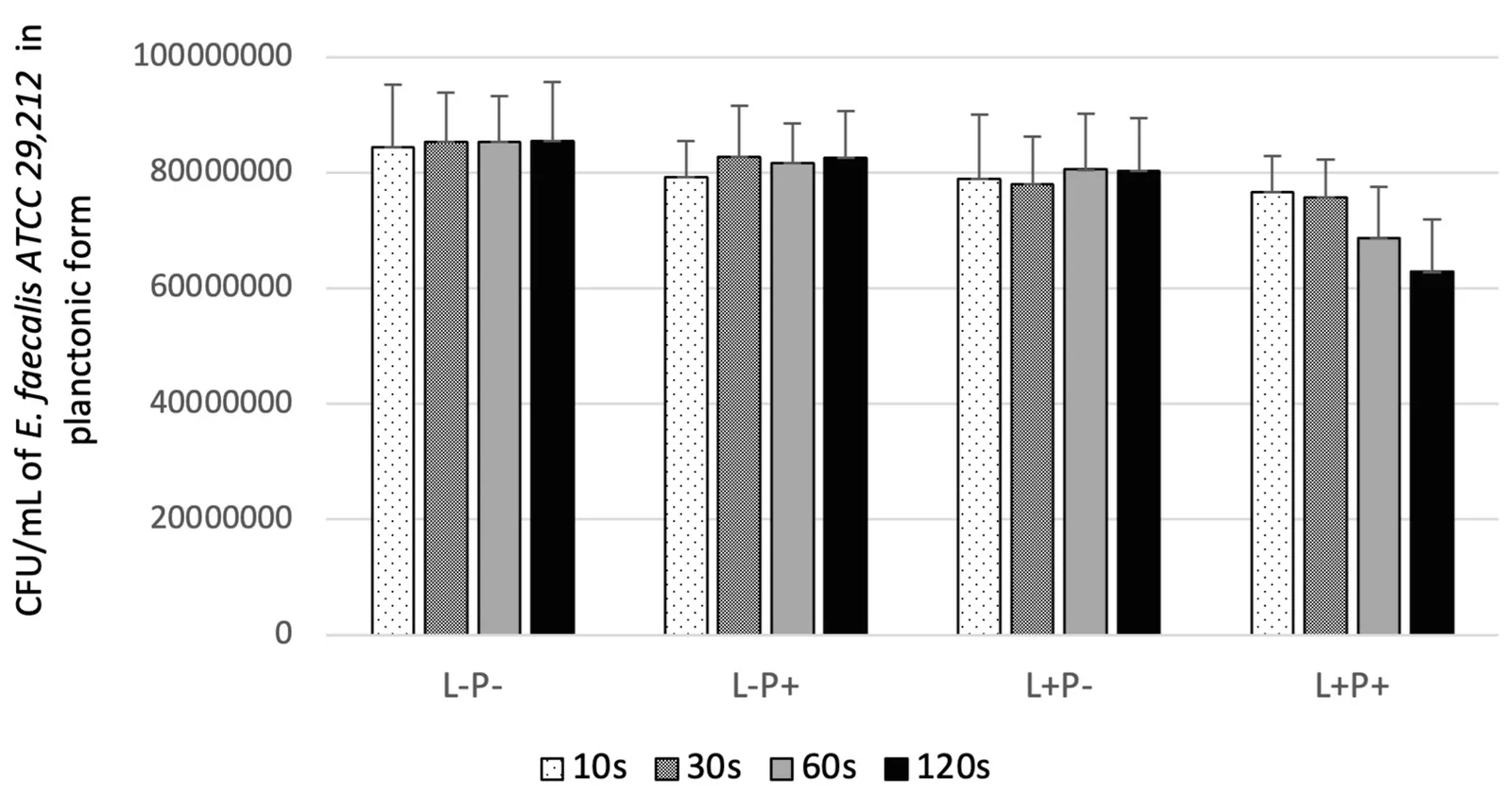
CFU/mL of planktonic *E. faecalis* ATCC 29,212 across irradiation times (10–120 s) for L−P−, L−P+, L+P−, and L+P+ groups, showing only a slight, time-dependent reduction in the combined L+P+ group.

*Enterococcus faecalis*. Reductions rose from 9.3% (0.042 log_10_) at 10 s to 26.5% (0.134 log_10_) at 120 s. The light × photosensitizer interaction did not reach significance at any individual time point in the two-way model (all *p* > 0.06), although the 60 s and 120 s conditions each showed significant reductions in L+P+ relative to control in direct pairwise comparison (*p* = 0.006 and *p* = 0.002, respectively; Figure 5), indicating a measurable but comparatively modest combined effect relative to the other species tested.

A summary of the results for each species is presented in Table 4 and Table 5 below.

**Table 4:** Effect of irradiation time on photodynamic inactivation of tested *Candida* species (450 nm, 400 mW, 15 min incubation, 100 µL photosensitizer).

Species	Irradiation Time	L−P− Control (CFU/mL, Mean ± SD)	L+P+ aPDT (CFU/mL, Mean ± SD)	Reduction	log_10_ Red.	Interaction F(1,20)	Interaction *p*
*C. albicans*	10 s	3.25 × 10^6^ ± 3.97 × 10^5^	2.21 × 10^6^ ± 3.80 × 10^5^	32.2%	0.169	2.71	0.115
	30 s	3.48 × 10^6^ ± 5.42 × 10^5^	1.91 × 10^6^ ± 3.12 × 10^5^	45.0%	0.260	3.77	0.067
	60 s	3.33 × 10^6^ ± 4.78 × 10^5^	1.71 × 10^6^ ± 2.00 × 10^5^	48.6%	0.289	7.61	0.012
	120 s	3.21 × 10^6^ ± 2.95 × 10^5^	1.49 × 10^6^ ± 2.26 × 10^5^	53.5%	0.333	29.51	<0.0001
*C. glabrata*	10 s	1.10 × 10^7^ ± 9.76 × 10^5^	7.35 × 10^6^ ± 1.34 × 10^6^	33.2%	0.175	8.68	0.008
	30 s	1.09 × 10^7^ ± 7.89 × 10^5^	7.30 × 10^6^ ± 9.06 × 10^5^	33.2%	0.175	14.19	0.0012
	60 s	1.09 × 10^7^ ± 1.36 × 10^6^	7.41 × 10^6^ ± 1.19 × 10^6^	32.3%	0.169	4.42	0.048
	120 s	1.09 × 10^7^ ± 1.48 × 10^6^	6.79 × 10^6^ ± 1.16 × 10^6^	37.9%	0.207	12.50	0.0021
*C. krusei*	10 s	2.27 × 10^6^ ± 4.30 × 10^5^	1.80 × 10^6^ ± 1.77 × 10^5^	20.8%	0.101	0.66	0.425
	30 s	2.33 × 10^6^ ± 3.13 × 10^5^	1.62 × 10^6^ ± 2.51 × 10^5^	30.4%	0.158	0.00	0.954
	60 s	2.44 × 10^6^ ± 4.59 × 10^5^	1.57 × 10^6^ ± 3.96 × 10^5^	35.8%	0.192	0.32	0.581
	120 s	2.25 × 10^6^ ± 3.19 × 10^5^	1.44 × 10^6^ ± 2.81 × 10^5^	35.9%	0.193	3.81	0.065

**Table 5:** Effect of irradiation time on photodynamic inactivation of tested bacterial species (450 nm, 400 mW, 15 min incubation, 50 µL photosensitizer).

Species	Irradiation Time	L−P− Control (CFU/mL, Mean ± SD)	L+P+ aPDT (CFU/mL, Mean ± SD)	Reduction	log_10_ Red.	Interaction F(1,20)	Interaction *p*
*S. aureus*	10 s	66.8 × 10^6^ ± 7.12 × 10^6^	57.5 × 10^6^ ± 5.85 × 10^6^	13.8%	0.065	0.01	0.926
	30 s	65.4 × 10^6^ ± 8.13 × 10^6^	54.1 × 10^6^ ± 5.10 × 10^6^	17.3%	0.083	0.57	0.459
	60 s	67.2 × 10^6^ ± 5.60 × 10^6^	43.4 × 10^6^ ± 10.96 × 10^6^	35.5%	0.190	6.67	0.018
	120 s	64.3 × 10^6^ ± 7.60 × 10^6^	34.3 × 10^6^ ± 7.53 × 10^6^	46.7%	0.274	17.14	0.0005
*E. faecalis*	10 s	84.4 × 10^6^ ± 10.81 × 10^6^	76.5 × 10^6^ ± 6.39 × 10^6^	9.3%	0.042	0.15	0.701
	30 s	85.3 × 10^6^ ± 8.50 × 10^6^	75.7 × 10^6^ ± 6.61 × 10^6^	11.4%	0.052	0.00	0.968
	60 s	85.3 × 10^6^ ± 7.87 × 10^6^	68.7 × 10^6^ ± 8.84 × 10^6^	19.5%	0.094	1.42	0.247
	120 s	85.5 × 10^6^ ± 10.23 × 10^6^	62.8 × 10^6^ ± 9.08 × 10^6^	26.5%	0.134	3.77	0.066

#### 3.3.2. Effect of Laser Output Power

*Candida albicans*. Reductions increased with power up to 200 mW (25.9%, 39.6%, and 43.6% at 50, 100, and 200 mW; 0.130, 0.219, and 0.248 log_10_), reaching a maximum of 53.5% (0.333 log_10_) at 400 mW. The light × photosensitizer interaction reached significance only at 400 mW (F(1,20) = 29.51, *p* < 0.0001), approaching but not reaching significance at 100 and 200 mW (*p* = 0.054 and *p* = 0.089, respectively).

*Candida glabrata*. Reductions were relatively stable across 50–200 mW (28.6–31.1%; 0.146–0.162 log_10_), rising to 37.9% (0.207 log_10_) at 400 mW. The light × photosensitizer interaction was significant at every power level tested.

*Candida krusei*. At 50 mW, no group differed significantly from any other (L+P+ vs. L−P−: *p* = 0.086; 22.7% reduction, 0.112 log_10_). At 100 and 200 mW, L+P+ was significantly lower than L−P− (*p* = 0.0072 and *p* = 0.0056, respectively; 30.6% and 31.7% reduction), reaching 35.9% (0.193 log_10_) at 400 mW. The light × photosensitizer interaction did not reach significance at any power level (all *p* > 0.06), mirroring the pattern observed in the irradiation-time series for this species.

*Staphylococcus aureus*. At 50 mW, no significant reduction was observed (8.2%; 0.037 log_10_; *p* = 0.323). Reductions became significant from 100 mW onward (20.2%, *p* = 0.018; 31.9%, *p* = 0.0071 at 200 mW), reaching a maximum of 46.7% (0.274 log_10_) at 400 mW, where the light × photosensitizer interaction was strongest (F(1,20) = 17.14, *p* = 0.0005).

*Enterococcus faecalis*. At 50 mW, no significant reduction was observed (8.4%; 0.038 log_10_; *p* = 0.170). Reductions became statistically significant from 100 mW onward (13.9%, *p* = 0.044; 18.1%, *p* = 0.016 at 200 mW), reaching a maximum of 26.5% (0.134 log_10_) at 400 mW. As in the irradiation-time series, the light × photosensitizer interaction term did not reach significance at any power level tested (all *p* > 0.06).

This is summarised in Table 6 and Table 7 below.

**Table 6:** Effect of laser output power on photodynamic inactivation of tested *Candida* species (450 nm, 120 s, 15 min incubation, 100 µL photosensitizer).

Species	Power	L−P− Control (Mean)	L+P+ (Mean)	Reduction	log_10_ Red.	*p* (L+P+ vs. L−P−)	Interaction F(1,20)	Interaction *p*
*C. albicans*	50 mW	3.37 × 10^6^	2.50 × 10^6^	25.9%	0.130	0.022	0.52	0.479
	100 mW	3.42 × 10^6^	2.07 × 10^6^	39.6%	0.219	0.0006	4.20	0.054
	200 mW	3.41 × 10^6^	1.93 × 10^6^	43.6%	0.248	0.0004	3.20	0.089
	400 mW	3.21 × 10^6^	1.49 × 10^6^	53.5%	0.333	<0.0001	29.51	<0.0001
*C. glabrata*	50 mW	1.11 × 10^7^	7.85 × 10^6^	29.1%	0.149	<0.0001	6.34	0.020
	100 mW	1.08 × 10^7^	7.47 × 10^6^	31.1%	0.162	0.0001	10.88	0.0036
	200 mW	1.08 × 10^7^	7.68 × 10^6^	28.6%	0.146	0.0008	6.53	0.019
	400 mW	1.09 × 10^7^	6.79 × 10^6^	37.9%	0.207	0.0003	12.50	0.0021
*C. krusei*	50 mW	2.35 × 10^6^	1.81 × 10^6^	22.7%	0.112	0.086	0.41	0.528
	100 mW	2.48 × 10^6^	1.72 × 10^6^	30.6%	0.159	0.0072	1.23	0.281
	200 mW	2.52 × 10^6^	1.72 × 10^6^	31.7%	0.166	0.0056	0.04	0.844
	400 mW	2.25 × 10^6^	1.44 × 10^6^	35.9%	0.193	0.0009	3.81	0.065

**Table 7:** Effect of laser output power on photodynamic inactivation of tested bacterial species (450 nm, 120 s, 15 min incubation, 50 µL photosensitizer).

Species	Power	L−P− Control (Mean)	L+P+ (Mean)	Reduction	log_10_ Red.	*p* (L+P+ vs. L−P−)	Interaction F(1,20)	Interaction *p*
*S. aureus*	50 mW	65.2 × 10^6^	59.9 × 10^6^	8.2%	0.037	0.323	0.07	0.800
	100 mW	67.3 × 10^6^	53.7 × 10^6^	20.2%	0.098	0.018	0.02	0.884
	200 mW	64.2 × 10^6^	43.7 × 10^6^	31.9%	0.167	0.0071	6.35	0.020
	400 mW	64.3 × 10^6^	34.3 × 10^6^	46.7%	0.274	<0.0001	17.14	0.0005
*E. faecalis*	50 mW	83.8 × 10^6^	76.7 × 10^6^	8.4%	0.038	0.170	0.00	0.997
	100 mW	84.7 × 10^6^	72.9 × 10^6^	13.9%	0.065	0.044	0.07	0.792
	200 mW	85.4 × 10^6^	70.0 × 10^6^	18.1%	0.086	0.016	0.30	0.589
	400 mW	85.5 × 10^6^	62.8 × 10^6^	26.5%	0.134	0.0023	3.77	0.066

### 3.4. Comparative Summary of Species Susceptibility

Under the optimized conditions identified in Stages I–III, all five tested species showed detectable reductions in the L+P+ group relative to controls, though the magnitude of these reductions was, in absolute microbiological terms, modest: maximum log_10_ reductions ranged from 0.134 to 0.333 across species (Table 8), corresponding to roughly 25–54% relative killing and falling below the ≥1 log_10_ reduction conventionally used as a benchmark for substantial antimicrobial activity. *C. albicans* showed the greatest reduction of any species tested (53.5%; 0.333 log_10_), followed by *S. aureus* (46.7%; 0.274 log_10_), *C. glabrata* (37.9%; 0.207 log_10_), *C. krusei* (35.9%; 0.193 log_10_), and *E. faecalis* (26.5%; 0.134 log_10_). In no experiment did laser irradiation alone or photosensitizer exposure alone produce a significant reduction relative to control, and a significant light × photosensitizer interaction was demonstrated for both *Candida* species with the strongest overall response (*C. albicans*, *C. glabrata*) and for *S. aureus* at higher irradiation doses, supporting a photodynamic-specific mechanism of action. For *C. krusei* and *E. faecalis*, reductions in L+P+ were consistently significant in direct pairwise comparison against control, but the two-way interaction term did not reach significance at the individual dose levels tested, indicating a comparatively weaker and less dose-separable combined effect in these two species.

Given the modest log_10_ reductions observed across all species, these results indicate measurable, statistically supported photodynamic activity under the planktonic, single-exposure conditions tested, which could serve as an adjunct to classical therapies rather than microbicidal or fungicidal killing sufficient to eradicate the tested inoculum. All experiments used ATCC reference strains in planktonic form; efficacy against clinical isolates or biofilm-embedded organisms was not assessed in this study.

## 4. Discussion

This systematic dose-optimization study demonstrates that antimicrobial photodynamic therapy using riboflavin 5′-phosphate (0.1%) as a photosensitizer activated by a 450 nm diode laser produces consistent and reproducible reductions in viable colony counts of *C. albicans*, *C. glabrata*, *C. krusei*, *S. aureus*, and *E. faecalis*. The observed antimicrobial effects were attributable exclusively to the photodynamic interaction between the photosensitizer and light, as neither laser irradiation alone nor photosensitizer exposure alone produced meaningful reductions in microbial viability across any of the tested species. These findings support the rejection of the null hypothesis and establish riboflavin 5′-phosphate-mediated aPDT as a promising broad-spectrum adjunctive approach for the treatment of both superficial fungal and bacterial infections.

### 4.1. Photosensitizer Selection and Formulation

The initial observation that European Pharmacopoeia-grade riboflavin at a maximum achievable concentration of 0.01% failed to produce antimicrobial effects, while riboflavin 5′-phosphate sodium salt hydrate at 0.1% demonstrated consistent activity against all tested species, highlights the critical importance of photosensitizer formulation and concentration regardless of the target organism. This finding aligns with previous reports demonstrating that riboflavin 5′-phosphate (FMN) exhibits superior water solubility and photochemical efficiency compared to riboflavin [14,15]. The enhanced performance of riboflavin 5′-phosphate may be attributed to its phosphate group, which increases aqueous solubility and facilitates more uniform distribution in the treatment medium [14]. Additionally, the 10-fold higher concentration achievable with riboflavin 5′-phosphate likely contributed to increased ROS generation and enhanced antimicrobial efficacy against both fungal and bacterial targets [12,16].

The photochemical properties of riboflavin 5′-phosphate make it particularly well-suited for broad-spectrum aPDT applications. Upon excitation by blue light, riboflavin 5′-phosphate generates both Type I and Type II ROS, with a singlet oxygen quantum yield of approximately 0.54 [13]. Studies employing fluorescent probes have demonstrated that riboflavin 5′-phosphate photolysis produces superoxide radical anions as the predominant Type I ROS, along with singlet oxygen via Type II mechanisms [14,15,17]. The relative contribution of these ROS to antimicrobial activity may vary depending on the target organism and microenvironment [17,23]. In the context of fungal inactivation, both singlet oxygen and superoxide radicals likely contribute to cell death through oxidative damage to the fungal cell membrane, mitochondria, and other cellular components [24,25,26]. Against Gram-positive bacteria such as *S. aureus* and *E. faecalis*, ROS-mediated damage to the cytoplasmic membrane, membrane proteins, and DNA represents the primary mechanism of inactivation, and the relatively accessible cell wall architecture of Gram-positive organisms is thought to facilitate photosensitizer interaction and ROS delivery compared to Gram-negative species [8,16].

### 4.2. Optimization of Pre-Irradiation Incubation Time

The identification of 15 min as the optimal pre-irradiation incubation time for *C. albicans* represents an important practical finding with likely relevance across the tested microbial panel. This incubation period allows sufficient time for photosensitizer uptake and distribution while maintaining clinical feasibility. The observed reduction in efficacy at 20 min, despite continued incubation, suggests that photosensitizer uptake may plateau or that cellular adaptive responses may begin to mitigate photosensitizer accumulation [19,26]. This finding is consistent with previous studies employing toluidine blue-mediated aPDT against *Candida* species, which identified optimal incubation times of approximately 10 min [27], and with antibacterial aPDT studies reporting comparable incubation optima for staphylococcal and enterococcal species.

The mechanism of photosensitizer uptake differs between fungal and bacterial cells. In fungi, uptake may involve both passive diffusion and active transport, with riboflavin transporters potentially facilitating photosensitizer accumulation in some species [12,19]. In Gram-positive bacteria, the absence of an outer membrane allows more direct interaction between the photosensitizer and the cytoplasmic membrane, which may reduce the incubation time required for effective photosensitizer loading [8,16]. The negatively charged phosphate group of riboflavin 5′-phosphate may limit penetration across both fungal cell membranes and the peptidoglycan layer of Gram-positive bacteria compared to cationic photosensitizers; however, the present study demonstrates that sufficient photosensitizer accumulation occurs within 15 min to achieve significant antimicrobial effects across all tested species [8,16].

### 4.3. Photosensitizer Volume and Concentration Effects

The observation that 100 µL of photosensitizer produced greater antimicrobial efficacy than 150 µL (53.5% vs. 46.8% reduction for *C. albicans*) is counterintuitive and warrants careful consideration. This finding suggests that photosensitizer concentration may exhibit a non-linear relationship with antimicrobial efficacy, potentially due to self-quenching effects at higher concentrations [6,28]. At elevated photosensitizer concentrations, inner filter effects may reduce light penetration through the treatment medium, limiting photon availability for photosensitizer activation in deeper layers of the microbial suspension [6]. Additionally, high photosensitizer concentrations may promote aggregation or self-quenching, reducing the quantum yield of ROS generation [6,28].

This concentration-dependent attenuation phenomenon is not unique to antifungal aPDT and has been reported with multiple photosensitizers in antibacterial contexts as well, reinforcing the principle that dose optimization, rather than simple maximization of photosensitizer concentration, is essential for effective aPDT regardless of the target organism [6]. The optimal photosensitizer concentration represents a balance between sufficient photosensitizer availability for ROS generation and avoidance of concentration-dependent quenching effects. For clinical applications targeting both fungal and bacterial infections, this finding suggests that moderate photosensitizer concentrations may be preferable to very high concentrations, potentially reducing treatment costs and minimizing the risk of photosensitizer-related adverse effects.

### 4.4. Irradiation Time and Fluence Optimization

The progressive improvement in antimicrobial efficacy with increasing irradiation time from 10 to 60–120 s, followed by a plateau in several species, demonstrates a dose–response relationship characteristic of photodynamic processes and consistent across both the fungal and bacterial targets examined [29,30]. The total light dose (fluence) delivered during treatment represents the product of irradiance (power density) and irradiation time, and both parameters influence aPDT outcomes [20,30]. At 400 mW output power with an applicator area of approximately 0.5 cm^2^, the irradiance was approximately 800 mW/cm^2^, yielding fluences ranging from 8 J/cm^2^ (10 s) to 96 J/cm^2^ (120 s).

Notably, while *C. albicans* and *C. krusei* showed efficacy plateaus beyond 60 s, both *S. aureus* and *E. faecalis* continued to demonstrate progressive reductions with irradiation times up to 120 s, with the most statistically significant antibacterial effects consistently observed at this maximum duration. This distinction suggests that bacterial species may require higher cumulative light doses to overcome endogenous antioxidant defenses or to achieve sufficient ROS-mediated membrane disruption, and that irradiation time is a particularly critical parameter in bacterial aPDT protocols.

The plateau in antifungal efficacy beyond 60 s may reflect photobleaching of the photosensitizer during prolonged irradiation, progressive oxygen depletion limiting Type II ROS production, or upregulation of cellular antioxidant defenses in response to oxidative stress [6,9,26,31]. The fluence values employed in this study (8–96 J/cm^2^) are consistent with those reported in other aPDT investigations. Studies of photodynamic therapy for actinic keratosis using aminolevulinic acid and blue light typically employ fluences of approximately 10 J/cm^2^, while antimicrobial aPDT protocols have utilized fluences ranging from 10 to 245 J/cm^2^ depending on the photosensitizer and target organism [20,30]. The relatively modest fluences required for riboflavin 5′-phosphate-mediated aPDT against the tested species suggest that this approach may be clinically feasible with short treatment times applicable to both fungal and bacterial infection scenarios.

### 4.5. Laser Output Power and Irradiance Effects

The finding that higher laser output power generally improved antimicrobial efficacy, with 400 mW producing the greatest reductions across all tested species, aligns with the fundamental principles of photodynamic therapy. Higher irradiance increases the rate of photosensitizer excitation and ROS generation, potentially overwhelming cellular antioxidant defenses and producing more rapid and complete microbial inactivation [29,30]. However, partial dose–response saturation at higher power levels was observed across multiple species, with 200 mW producing results comparable to 400 mW for *C. albicans*, and with similar trends emerging for the bacterial strains at sub-maximal power settings.

This saturation effect may reflect oxygen depletion becoming rate-limiting for Type II ROS production at very high irradiance, as molecular oxygen is consumed faster than it can be replenished by diffusion, as well as accelerated photosensitizer photobleaching at higher power limiting total ROS production over the treatment period [6,31]. The pattern was broadly consistent across both fungal and bacterial species, suggesting that the underlying photophysical constraints on dose–response are organism-independent. From a clinical perspective, the ability to achieve substantial antimicrobial effects at moderate power levels is advantageous, as it reduces the risk of thermal damage to surrounding tissues and allows for the use of less expensive, lower-power light sources in both dental and dermatological applications.

### 4.6. Species-Specific Susceptibility Patterns

The differential susceptibility of the five tested species to riboflavin 5′-phosphate-mediated aPDT provides important insights into the potential clinical applications of this approach. Among the *Candida* species, *C. albicans* demonstrated the greatest susceptibility, with reductions of up to 53.5% under optimized conditions, while *C. krusei* and *C. glabrata* showed more moderate reductions (35.9% and 37.9%, respectively). Among the bacterial strains, *S. aureus* demonstrated greater susceptibility than *E. faecalis* across most tested parameter combinations, with statistically significant reductions observed at shorter irradiation times and lower power levels compared to the enterococcal strain.

The differential susceptibility between the two bacterial species may reflect intrinsic differences in cell wall thickness and composition, the efficiency of endogenous antioxidant systems, and the relative permeability of the cell envelope to ROS [8,9]. *E. faecalis* is known for robust stress tolerance mechanisms, including the expression of catalase, peroxidases, and other ROS-scavenging enzymes that may partially mitigate the oxidative damage induced by aPDT [9]. *S. aureus*, while similarly equipped with antioxidant defenses, may be more susceptible to ROS-mediated membrane disruption due to differences in membrane fatty acid composition and carotenoid content between strains [8,16]. These species-specific differences reinforce the importance of tailoring irradiation parameters to the target organism rather than applying a universal aPDT protocol.

Among the *Candida* species, *C. glabrata*, which exhibited the highest baseline colony counts and the lowest relative reduction, is known for its intrinsic reduced susceptibility to azole antifungals and robust stress response mechanisms [2,3]. Its enhanced antioxidant defenses, including higher catalase and superoxide dismutase activity compared to *C. albicans*, likely contribute to its relative resistance to oxidative stress-based therapies [9,26]. *C. krusei*, despite its intrinsic fluconazole resistance, demonstrated intermediate susceptibility to aPDT, consistent with previous reports showing variable but meaningful sensitivity of this species to photodynamic inactivation depending on the photosensitizer employed [18,19]. The present findings suggest that riboflavin 5′-phosphate-mediated aPDT may offer a valuable adjunctive strategy for both azole-resistant fungal infections and antibiotic-resistant bacterial infections, particularly in superficial or localized clinical presentations where direct light application is feasible.

### 4.7. Mechanism of Antimicrobial Action

The consistent finding that neither laser irradiation alone nor riboflavin 5′-phosphate alone produced meaningful reductions in viable counts across any of the five tested species, fungal or bacterial, is among the most important observations of this study. It confirms that the antimicrobial effects observed in the L+P+ group were attributable exclusively to the photodynamic mechanism, ruling out dark toxicity of the photosensitizer and photothermal effects of the laser as confounding factors [1,8]. This mechanistic specificity was reproducible across organisms with markedly different cell wall architectures, from the thick, ergosterol-containing fungal cell membrane of *Candida* spp. to the peptidoglycan-rich envelope of Gram-positive bacteria, suggesting that the photodynamic mechanism is sufficiently non-specific in its molecular targets to operate effectively across these structural differences. The antimicrobial mechanism of riboflavin 5′-phosphate-mediated aPDT involves the generation of multiple ROS that cause oxidative damage through both Type I and Type II photochemical pathways [5,7]. Type II reactions produce singlet oxygen, which has a short lifetime in aqueous solution (approximately 3.7 µs) but is highly reactive with unsaturated lipids, proteins, and nucleic acids [6,13]. Type I reactions generate superoxide radical anions, which dismutate to form hydrogen peroxide and can subsequently react with transition metals to produce hydroxyl radicals via Fenton chemistry [5,9]. Both pathways contribute to the antimicrobial effect, and the relative dominance of each may vary depending on local oxygen availability, photosensitizer concentration, and the biochemical environment of the target cell [17,23].

In fungal cells, ROS-mediated damage targets the cell membrane, particularly its ergosterol-rich lipid bilayer, as well as mitochondria and other organelles [24,25,26]. Lipid peroxidation of membrane phospholipids increases permeability and compromises cellular integrity, while mitochondrial damage disrupts energy metabolism and may activate apoptotic cascades [26,32]. The multi-target nature of this oxidative damage is consistent with the absence of photodynamic resistance development reported in the literature and observed indirectly in the present study through the susceptibility of both azole-resistant *C. glabrata* and intrinsically fluconazole-resistant *C. krusei* to aPDT [1,8]. In *S. aureus* and *E. faecalis*, the primary targets of ROS-mediated damage are the cytoplasmic membrane, membrane-associated proteins involved in energy transduction and transport, and DNA [8,9,17]. Gram-positive bacteria lack an outer membrane, which means that photosensitizer molecules can interact directly with the cytoplasmic membrane without the need to traverse a permeability barrier, potentially facilitating efficient ROS delivery to critical cellular targets [8,16]. Studies employing specific ROS scavengers in antibacterial aPDT have confirmed that both singlet oxygen and superoxide radicals contribute to bacterial killing, with their relative importance depending on the experimental conditions and the target organism [17,23]. The present results are consistent with these mechanistic data: significant reductions in *S. aureus* viability were observed at shorter irradiation times than for *E. faecalis*, which may partly reflect differences in membrane composition, carotenoid content, and ROS-scavenging capacity between the two species [8,9,16].

### 4.8. Clinical Implications and Translational Potential

The findings of this study have implications for the potential clinical application of riboflavin 5′-phosphate-mediated aPDT across a range of superficial and localized infections caused by both fungi and Gram-positive bacteria. The optimised parameters identified that 15-min pre-irradiation incubation, 100 µL photosensitizer volume for *Candida* spp. and 50 µL for bacterial species, with irradiation times of 60–120 s and output powers of 200–400 mW, provide a starting framework for the development of standardized clinical protocols. The relatively short total treatment time (approximately 16–17 min including incubation and irradiation) and the use of a non-toxic, naturally occurring photosensitizer supports the clinical feasibility and patient tolerability of this approach. For fungal infections, riboflavin 5′-phosphate-mediated aPDT is particularly relevant to the management of superficial and mucosal *Candida* infections, including oral candidiasis, denture stomatitis, angular cheilitis, vulvovaginal candidiasis, and cutaneous candidiasis [1,11,33]. These conditions are well-suited to topical photosensitizer delivery and direct light application, and the limited penetration depth of 450 nm blue light (approximately 1–2 mm) is adequate for treating superficial mucosal lesions [20,22]. Clinical studies of aPDT for oral erythematous candidiasis have demonstrated clinically meaningful reductions in *Candida* colony counts and objective improvement in lesion severity with minimal adverse effects, supporting the translational relevance of the in vitro parameters established here [33].

For bacterial infections, the bactericidal activity demonstrated against *S. aureus* and *E. faecalis* in the present study is directly relevant to several clinical scenarios amenable to local light delivery. *S. aureus* is a leading causative agent of wound infections, impetigo, and infected surgical sites, while *E. faecalis* is the predominant pathogen in persistent endodontic infections and is frequently isolated from infected root canals and periapical lesions [9]. Both pathogens are also implicated in periodontal disease and peri-implant infections, settings in which aPDT has been investigated as an adjunct to conventional mechanical debridement [34]. The efficacy of riboflavin 5′-phosphate, already the subject of investigation in periodontal aPDT [34], against these two species under systematically optimised conditions strengthens the evidence base for its clinical use in these contexts.

The safety profile of riboflavin 5′-phosphate is a particular advantage shared across all potential clinical applications. As an essential water-soluble vitamin (vitamin B_2_), riboflavin is non-toxic at therapeutic concentrations and is well tolerated by human tissues [11,34]. Studies examining the cytotoxicity of riboflavin-mediated PDT on normal human cells have demonstrated minimal toxicity to fibroblasts, keratinocytes, and other cell types at photosensitizer concentrations and light doses that effectively kill both fungal and bacterial cells [16,34,35]. This selective toxicity toward microbial over mammalian cells likely reflects differences in photosensitizer uptake efficiency, endogenous antioxidant capacity, and cellular repair mechanisms [8,16]. The absence of tissue staining, a significant cosmetic limitation of phenothiazinium dyes such as methylene blue and toluidine blue, represents an additional practical advantage, particularly for oral and mucosal applications [11,34].

The emergence of multidrug-resistant pathogens further strengthens the case for aPDT as an adjunctive strategy. For *Candida* infections, the growing prevalence of azole-resistant *C. albicans* and intrinsically resistant *C. glabrata* and *C. krusei* has created an urgent therapeutic gap that conventional antifungals are increasingly unable to fill [2,3,36]. For bacterial infections, methicillin-resistant *S. aureus* (MRSA) and vancomycin-resistant *Enterococcus* (VRE) represent analogous challenges in both community and healthcare settings [8,9]. Because the photodynamic mechanism operates through multi-target oxidative stress rather than through specific molecular targets susceptible to conventional resistance mechanisms, aPDT is theoretically effective regardless of the conventional resistance profile of the target organism [1,8]. The present findings support this principle: both azole-resistant fungal species and Gram-positive bacteria known for robust stress-response systems were susceptible to riboflavin 5′-phosphate-mediated aPDT under optimised conditions.

### 4.9. Comparison with Other Photosensitizers

The antimicrobial efficacy of riboflavin 5′-phosphate-mediated aPDT observed in the present study can be contextualised within the broader literature on photosensitizers evaluated against both *Candida* species and Gram-positive bacteria. Phenothiazinium dyes—primarily methylene blue and toluidine blue—are among the most extensively studied photosensitizers for antimicrobial aPDT against both fungal and bacterial targets [27,29]. A recent study employing toluidine blue activated by a 635 nm diode laser reported significant reductions in *C. albicans*, *C. glabrata*, and *C. krusei* viability, as well as against *S. aureus*, under similar experimental conditions and comparable parameter ranges (10-min incubation, 400 mW, 120 s irradiation) [27]. The antifungal reductions achieved with toluidine blue in that study were broadly comparable to those observed here with riboflavin 5′-phosphate, suggesting similar photodynamic potency against planktonic *Candida* cells. However, toluidine blue and methylene blue are associated with visible tissue staining and, in some formulations, measurable dark toxicity, which limits their applicability to mucosal surfaces and open wounds [11,27].

Against *S. aureus*, cationic riboflavin derivatives have been shown to produce substantially more potent photodynamic inactivation than the anionic riboflavin 5′-phosphate used in the present study, achieving near-complete killing of multiresistant strains including MRSA at relatively low photosensitizer concentrations [16]. The superior antibacterial efficacy of cationic riboflavin derivatives is attributed to enhanced electrostatic interaction with the negatively charged bacterial cell surface, facilitating greater photosensitizer uptake and more efficient ROS delivery [16]. While the neutral-to-anionic character of riboflavin 5′-phosphate limits its cell-surface binding compared to cationic congeners, the consistent and statistically significant bactericidal effects against both *S. aureus* and *E. faecalis* observed in the present study demonstrate that effective antibacterial activity is achievable without cationic modification, albeit at higher fluences. Studies employing riboflavin 5′-phosphate photolysis specifically against *S. aureus* have reported reductions exceeding 95% under optimised conditions using violet or blue light [15], suggesting that the modest reductions achieved here may partly reflect the fixed inoculum density and the particular optical geometry of the microplate assay rather than an intrinsic ceiling of photosensitizer potency. For *E. faecalis*, aPDT efficacy data using riboflavin-based photosensitizers are more limited in the published literature, but studies characterising the oxidative damage profile of riboflavin 5′-phosphate against MRSA and related Gram-positive pathogens have identified DNA strand breaks, membrane lipid peroxidation, and protein carbonylation as principal damage endpoints, with both Type I and Type II ROS contributing to the overall antibacterial effect [17]. The present study extends this mechanistic framework to *E. faecalis*, demonstrating that irradiation-time-dependent reductions in viability follow a pattern consistent with progressive ROS-mediated damage accumulation.

Against *Candida* species other than *C. albicans*, comparisons with alternative photosensitizers are instructive. Rose bengal, activated by green light, has demonstrated potent antifungal activity against drug-resistant *C. albicans* in vitro [37,38,39,40,41,42], but its significant dark toxicity and photodegradation profile limit clinical utility. Studies employing Photofrin against *Candida* species, including *C. krusei*, have demonstrated susceptibility comparable to *C. albicans* at photosensitizer concentrations above 3 µg/mL [19], consistent with the intermediate susceptibility of *C. krusei* observed in the present study. Natural photosensitizers, including curcumin derivatives and hypericin, have shown promising antifungal activity with favorable biocompatibility profiles [11,24]; hypericin-mediated aPDT has been reported to achieve *Candida* biofilm reductions comparable to those of riboflavin 5′-phosphate in planktonic form [11]. Taken together, riboflavin 5′-phosphate occupies a practical position in the photosensitizer landscape: its efficacy against both fungal and Gram-positive bacterial targets is moderate rather than maximal when compared with optimised cationic or porphyrin-based photosensitizers, but its exceptional safety profile, absence of tissue staining, low cost, aqueous solubility, and activation by inexpensive blue-light sources make it a highly translatable candidate for broad-spectrum clinical aPDT applications. The present study provides the parameter-specific data necessary to evaluate and further develop this potential.

### 4.10. Limitations and Future Directions

Several limitations of this study should be acknowledged. First, the experiments were conducted using planktonic *Candida* cells in vitro, which may not fully represent the complexity of *Candida* infections in vivo. *Candida* biofilms, which are commonly encountered in clinical infections such as denture stomatitis and catheter-associated candidiasis, exhibit enhanced resistance to antimicrobial agents compared to planktonic cells [10,11,38]. Future studies should evaluate the efficacy of riboflavin 5′-phosphate-mediated aPDT against *Candida* biofilms, as biofilm eradication represents a significant clinical challenge [10,11].

Second, the study employed standard reference strains (ATCC) rather than clinical isolates. While reference strains provide reproducibility and allow for standardized comparisons, clinical isolates may exhibit different susceptibility patterns due to genetic diversity and prior antifungal exposure [10,36]. Future investigations should include clinical isolates of *Candida* species, including azole-resistant strains, to better assess the clinical applicability of this approach.

Third, the maximum reduction in viable colony counts achieved in this study was approximately 53% for *C. albicans*, which represents a significant but incomplete antimicrobial effect. Therefore, these findings suggest that the proposed approach should be considered an adjunctive therapy supporting conventional antimicrobial treatment. Strategies to enhance efficacy should be explored, including combination with conventional antifungal agents, use of photosensitizer delivery systems such as nanoparticles to enhance cellular uptake, or sequential application of multiple aPDT sessions [36,38]. Combined curcumin–riboflavin photosensitizer systems have already been evaluated in vitro against *Candida* spp. and *S. aureus* using 450 nm diode laser irradiation [39]; such formulations are also commercially available for oral-health applications (e.g., QroxB2, CMS Dental, Roslev, Denmark). Fourth, the study did not include in vivo experiments to assess efficacy and safety in animal models of candidiasis. In vivo studies are essential for evaluating tissue penetration, photosensitizer distribution, host immune responses, and potential adverse effects in a physiologically relevant context [29,40]. Previous studies of aPDT for oral candidiasis in murine models have demonstrated clinical efficacy and safety, providing a foundation for future investigations of riboflavin 5′-phosphate-mediated aPDT in vivo [40,41,42].

Only ATCC reference strains were used, that clinical isolates (including azole-resistant *Candida*, MRSA, VRE) may show different susceptibility, and that this is a priority for future work.

Finally, the study did not investigate the mechanisms underlying the species-specific differences in susceptibility to aPDT. Future research should employ mechanistic approaches, including measurement of intracellular ROS levels, assessment of antioxidant enzyme activity, evaluation of photosensitizer uptake, and analysis of cell death pathways, to elucidate the factors determining *Candida* susceptibility to riboflavin 5′-phosphate-mediated aPDT [17,24,25,26].

## 5. Conclusions

This study systematically characterises riboflavin 5′-phosphate-mediated antimicrobial photodynamic therapy (aPDT) using a 450 nm diode laser against fungal and bacterial pathogens. Photodynamic treatment (L+P+) produced consistent, significant reductions across all species tested, *C. albicans, C. glabrata, C. krusei, S. aureus*, and *E. faecalis*, while laser or photosensitiser alone was ineffective, confirming the effect is strictly photodynamic. Efficacy was governed by a narrow therapeutic window. A 15-min pre-irradiation incubation was optimal, while increasing photosensitiser volume beyond 100 µL reduced activity, likely due to optical attenuation. Irradiation time was the primary determinant: *C. albicans and C. krusei* plateaued at 60 s, *C. glabrata* required 120 s, and bacterial reductions accumulated up to 120 s, with *S. aureus* responding earlier than *E. faecalis*. Laser power had a secondary role, with saturation observed at higher outputs, indicating high-power devices are not required. Maximal microbial reductions reached 53.5% for *C. albicans* and >46% for *S. aureus*, suggesting that aPDT is most appropriate as an adjunctive rather than stand-alone therapy. Its mechanism is independent of conventional drug targets, making it relevant against resistant strains. The protocol is clinically feasible, using a non-toxic, inexpensive photosensitiser and compact visible-light laser in approximately 16–17 min. However, translation to biofilm models and in vivo systems is essential, as photosensitiser penetration and microbial metabolic state may limit efficacy. Riboflavin 5′-phosphate aPDT demonstrates reproducible, species-specific broad-spectrum activity under optimised conditions, with translational potential that requires further validation in complex infection models.

## Figures and Tables

**Table 1:** Working densities of microbial suspensions in individual variants of Stage II.

Experimental Variant	Working Density	Suspension Volume per Well	PS Volume per Well	Total Volume per Well	Total Microbial Cells per Well
Variant A (50 µL PS)	3 × 10^8^ CFU/mL	200 µL	50 µL	250 µL	6 × 10^7^ CFU
Variant B (100 µL PS)	4 × 10^8^ CFU/mL	150 µL	100 µL	250 µL	6 × 10^7^ CFU
Variant C (150 µL PS)	6 × 10^8^ CFU/mL	100 µL	150 µL	250 µL	6 × 10^7^ CFU

Total microbial cells per well = working density (CFU/mL) × suspension volume (mL); e.g., Variant A: 3 × 10^8^ CFU/mL × 0.2 mL = 6 × 10^7^ CFU.

**Table 2:** Effect of pre-irradiation incubation time on photodynamic inactivation of *Candida albicans* (450 nm, 400 mW, 60 s, 50 µL photosensitizer).

Incubation Time	L−P− Control (CFU/mL, Mean ± SD, n = 3)	L+P+ aPDT (CFU/mL, Mean ± SD, n = 5)	Reduction vs. Control	log_10_ Reduction	*p*-Value
1 min	2.88 × 10^6^ ± 4.18 × 10^5^	2.18 × 10^6^ ± 4.75 × 10^5^	24.2%	0.120	0.082
5 min	2.84 × 10^6^ ± 5.38 × 10^5^	2.30 × 10^6^ ± 4.41 × 10^5^	18.9%	0.091	0.174
10 min	3.45 × 10^6^ ± 4.39 × 10^5^	2.32 × 10^6^ ± 5.24 × 10^5^	32.8%	0.173	0.021
15 min	2.72 × 10^6^ ± 4.00 × 10^5^	1.68 × 10^6^ ± 2.81 × 10^5^	38.2%	0.209	0.0047
20 min	3.12 × 10^6^ ± 8.94 × 10^5^	2.67 × 10^6^ ± 3.77 × 10^5^	14.4%	0.067	0.346
30 min	3.48 × 10^6^ ± 1.74 × 10^5^	2.22 × 10^6^ ± 4.04 × 10^5^	36.3%	0.196	0.0024

CFU/mL calculated as raw colony count × 4.0 × 10^4^, per Section 2.6.

**Table 3:** Effect of riboflavin 5′-phosphate volume on photodynamic inactivation of *Candida albicans* (450 nm, 400 mW, 60 s, 15 min incubation).

Photosensitizer Volume	L−P− Control (CFU/mL, Mean ± SD)	L+P+ aPDT (CFU/mL, Mean ± SD)	Reduction vs. Control	log_10_ Reduction
50 µL	3.23 × 10^6^ ± 4.14 × 10^5^	1.79 × 10^6^ ± 2.90 × 10^5^	44.5%	0.256
100 µL	4.00 × 10^6^ ± 3.70 × 10^5^	1.86 × 10^6^ ± 2.80 × 10^5^	53.5%	0.333
150 µL	4.26 × 10^6^ ± 6.80 × 10^5^	2.27 × 10^6^ ± 4.80 × 10^5^	46.8%	0.274

One-way ANOVA across volumes: F(2,15) = 2.05, *p* = 0.163 (n.s.).

**Table 8:** Optimal treatment parameters and maximum antimicrobial effect achieved for each tested species.

Species	Optimal Incubation Time	Optimal PS Volume	Optimal Irradiation Time	Optimal Power	Max. % Reduction	Max. log_10_ Reduction
*C. albicans* ATCC 10231	15 min	100 µL	120 s	400 mW	53.5%	0.333
*C. glabrata* ATCC 66032	15 min	100 µL	120 s	400 mW	37.9%	0.207
*C. krusei* ATCC 14243	15 min	100 µL	120 s	400 mW	35.9%	0.193
*S. aureus* ATCC 29213	15 min	50 µL	120 s	400 mW	46.7%	0.274
*E. faecalis* ATCC 29212	15 min	50 µL	120 s	400 mW	26.5%	0.134

## Data Availability

The data presented in this study are available on request from the corresponding author.
